# Giant peripheral ossifying fibroma with coincidental squamous cell carcinoma: a case report

**DOI:** 10.1186/s13256-021-03187-5

**Published:** 2021-12-19

**Authors:** Takeshi Karube, Kanako Munakata, Yuka Yamada, Yuta Yasui, Shosuke Yajima, Nobuyuki Horie, Hiromasa Kawana, Shuji Mikami, Taneaki Nakagawa, Seiji Asoda

**Affiliations:** 1 Department of Dentistry and Oral Surgery, Keio University School of Medicine, 35 Shinanomachi, Shinjuku-ku, Tokyo, Japan; 2 Department of Oral and Maxillofacial Implantology, Kanagawa Dental University, Yokosuka, Japan; 3 Division of Diagnostic Pathology, Keio University School of Medicine, Tokyo, Japan

**Keywords:** Peripheral ossifying fibroma, Oral squamous cell carcinoma

## Abstract

**Background:**

Peripheral ossifying fibroma is an inflammatory or reactive hyperplasia of the gingiva that is usually small. It is formed by hard tissue in fibrous tissue, and the name “neoplastic lesion” has tended to be used frequently in Europe and America. Clinically, peripheral ossifying fibromas are painless, solitary, exophytic, sessile, or pedunculated and more frequently found in females than in males. To the best of our knowledge, there have been no reports of malignant cases. We herein report the case of giant peripheral ossifying fibroma with squamous cell carcinoma.

**Case presentation:**

The patient was an 83-year-old Japanese woman who visited our hospital with a gingival massive mass. She was referred to us for an examination and treatment because it was difficult to perform tracheal intubation for surgery of sigmoid colon cancer at another hospital. The mass measured 83 × 58 × 35 mm, and it protruded to the extra-oral region from the right maxillary premolar alveolar region. Panoramic X-ray revealed the shadow of the mass in the right maxillary premolar region, which included some hard tissue. Computed tomography showed scattering calcified images in the mass. Magnetic resonance imaging was not performed because she had vertebral artery clips and screws in her forehead. Given the above findings, we performed a biopsy under local anesthesia. However, we were unable to diagnose absolutely whether the dysplastic squamous epithelia were pseudocarcinomatous hyperplasia of the gingiva or well-differentiated squamous cell carcinoma. Therefore, tumor resection was performed under general anesthesia. The histopathological diagnosis was peripheral ossifying fibroma with coincidental squamous cell carcinoma. There have been no signs of recurrence during follow-up as of 2 years after surgery.

**Conclusions:**

The etiology of giant peripheral ossifying fibroma with squamous cell carcinoma is still not definite. Therefore, careful observation is necessary. It needs to be examined by accumulation of more cases in the future. We herein report the case of giant peripheral ossifying fibroma coincidental squamous cell carcinoma.

## Background

Peripheral ossifying fibroma (POF) is an inflammatory or reactive hyperplasia of the gingiva and presents clinically as a painless, slowly growing mass. POF occurs more frequently in females than in males [1–3], especially female patients in the second or third decade of life [3, 4], and is more common in the anterior maxilla than in other locations [5, 6]. POFs are generally smaller than < 2 cm in size [7–9]. However, larger lesions have been rarely reported in the literature [8, 10]. Notably, there have been no reports of POF with coincidental squamous cell carcinoma. We herein report a long-term case of giant POF with coincidental squamous cell carcinoma.

## Case presentation

**Fig. 1.**
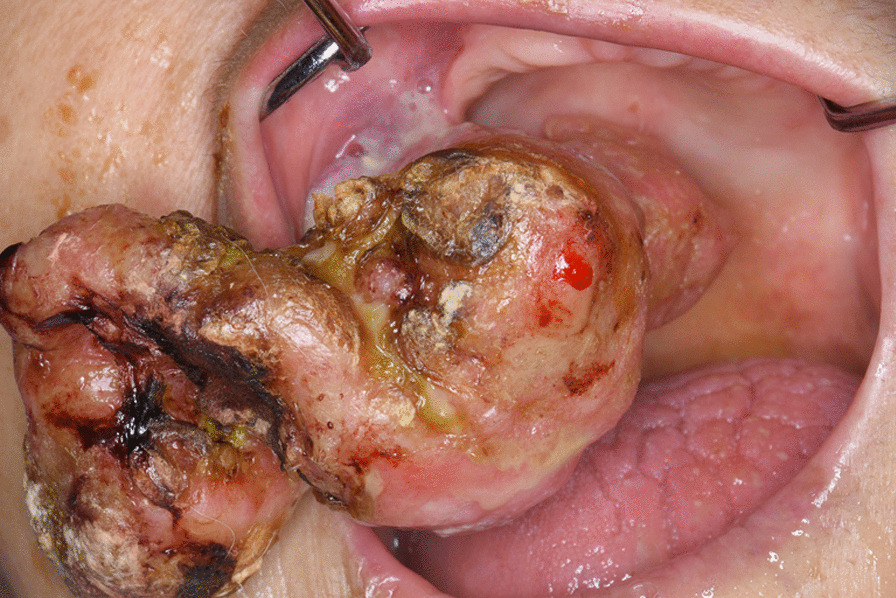
Intra- and extra-oral findings. A massive pedunculated mass in the right maxillary premolar region is observed. It protrudes to the extra-oral region from the right maxillary premolar alveolar region

In 2017, an 83-year-old Japanese woman presented to our department with a chief complaint of a mass in her right maxillary premolar region. She had initially noticed a painless mass in her right maxillary premolar region in 2002. After she had first noticed of the mass, it grew gradually in size, but she sought no treatment for it. She was referred to us for examination and treatment because it was difficult to perform tracheal intubation for surgery of sigmoid colon cancer at another hospital. Her history included sigmoid colon cancer, subarachnoid hemorrhaging, bronchitic asthma, and cardiac insufficiency. Regarding the intra- and extra-oral findings, a massive pedunculated mass in the right maxillary premolar region measuring 83 × 58 × 35 mm was palpable (Fig. 1). Furthermore, it covered the front of the right palate, and it protruded to the extra-oral region from the right maxillary premolar alveolar region. Its surface was almost entirely smooth, and some erosions and ulcerations were seen. It was elastic and hard and showed no tenderness on palpation. There was no palpable regional lymphadenopathy, and a laboratory examination revealed no abnormal values.

**Fig. 2.**
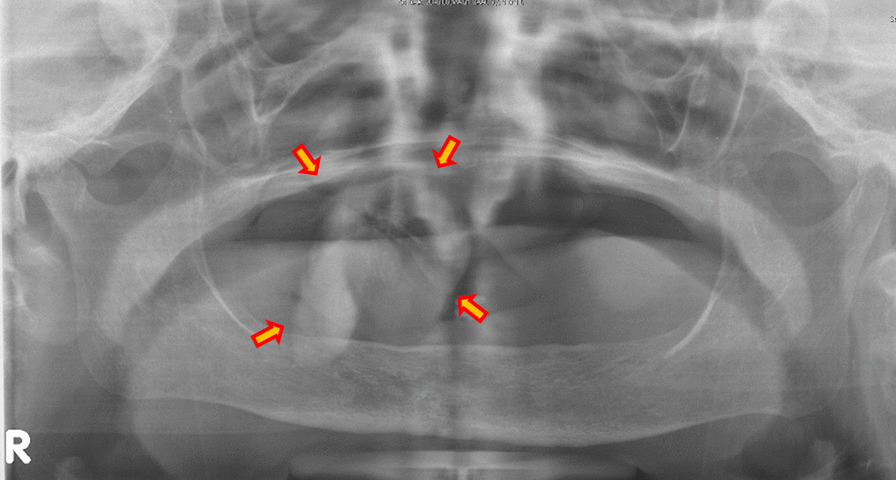
Panorama X-ray photograph revealing the shadow of the mass in the right maxillary premolar region, which includes some hard tissues (arrows)

**Fig. 3.**
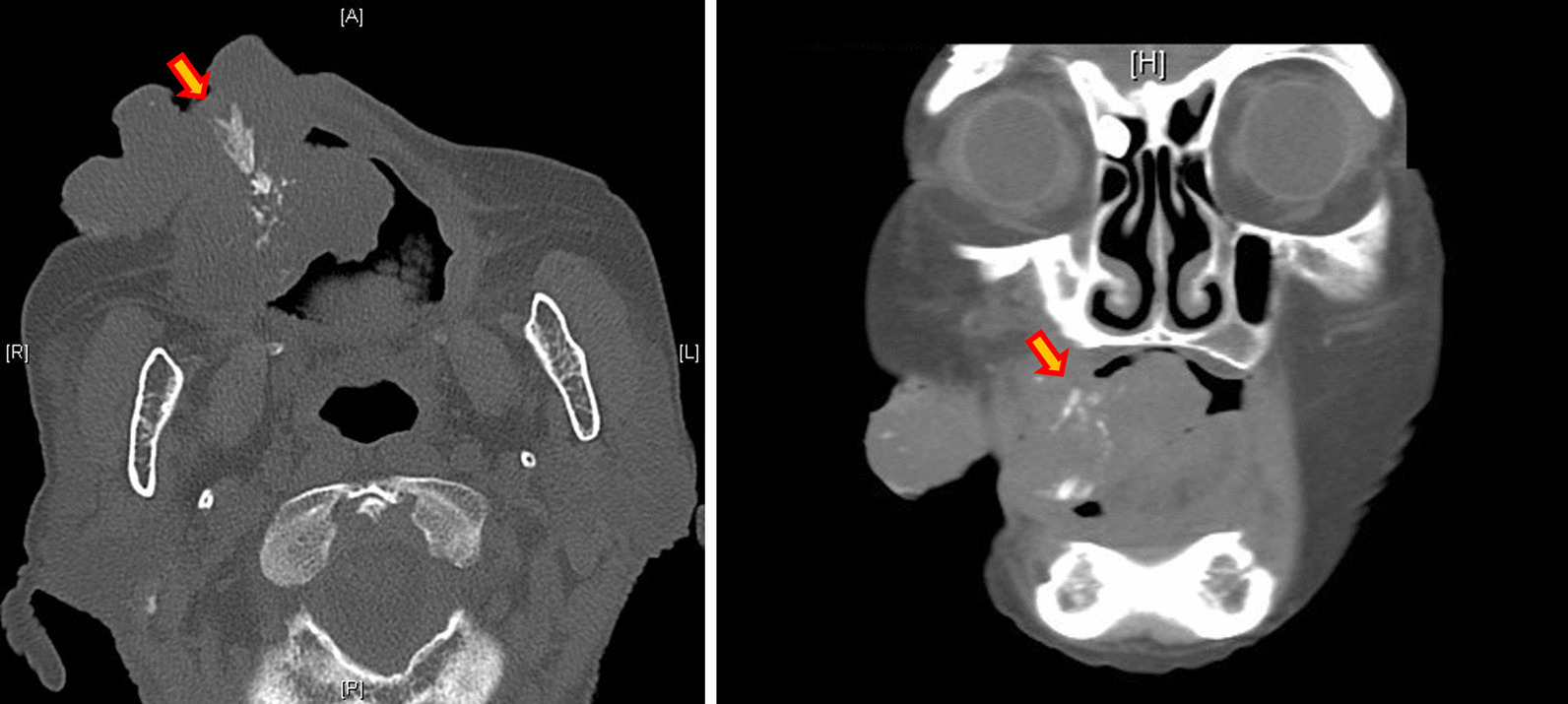
Computed tomography showing scattering calcified images in the mass (arrows)

**Fig. 4.**
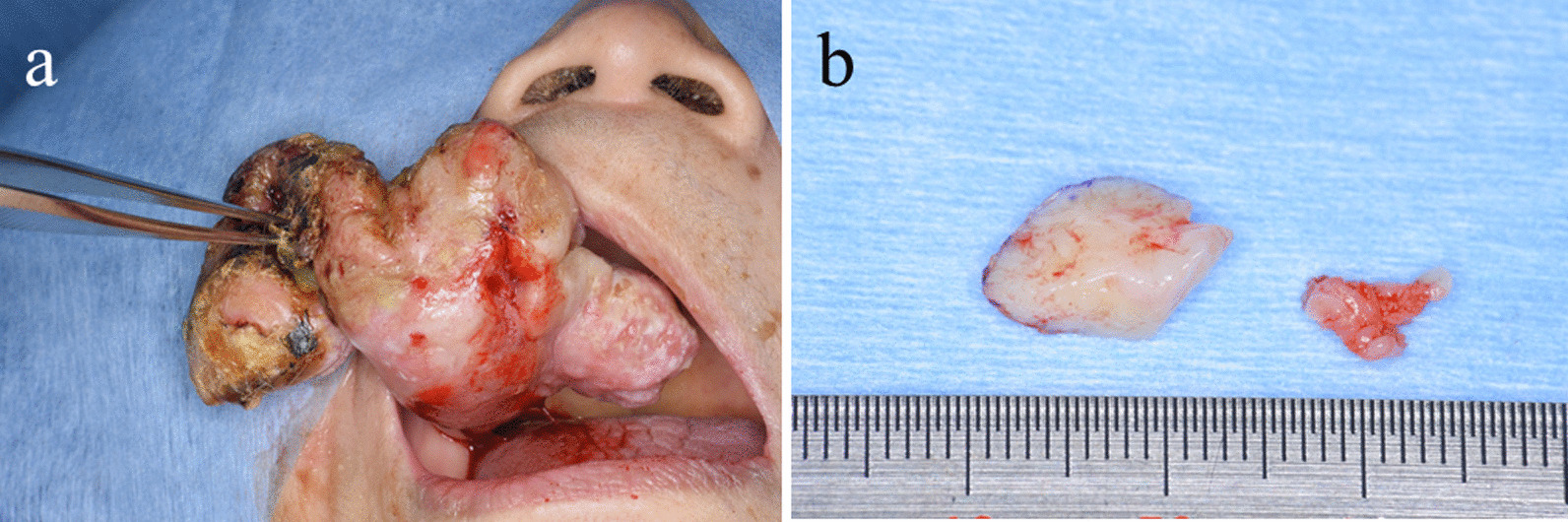
Operative findings from the biopsy. **a** Extraoperative photograph, **b** excised specimen

Panoramic X-ray revealed the shadow of the mass in the right maxillary premolar region, which included some hard tissue (Fig. 2). Computed tomography (CT) showed scattering calcified images in the mass (Fig. 3). Magnetic resonance imaging was not performed because she had vertebral artery clips and screws in her forehead. Given the above findings, we suspected benign gingival tumor in the right maxillary premolar region and performed a biopsy under local anesthesia (Fig. 4a, b).

**Fig. 5.**
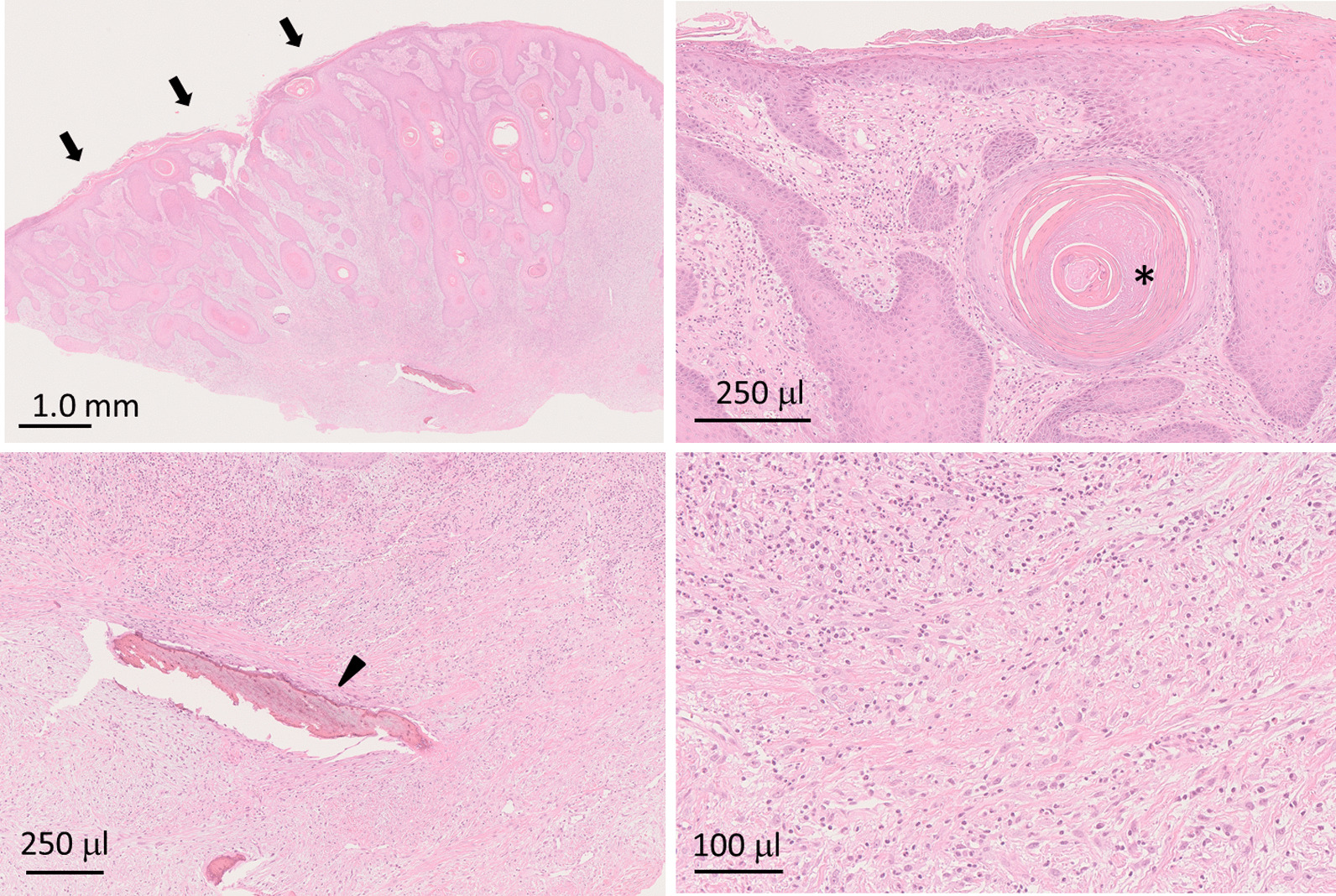
Histopathological findings (hematoxylin–eosin stain). The biopsy specimen was composed of dysplastic spindle-shaped epithelia (arrows) and stromal tissue. Some ossification (arrow head) and cancerous pearl formation (asterisk) are observed. High-magnification view of stromal tissue revealed spindle-shaped stromal cells with collagenous fiber (lower right figure)

**Fig. 6.**
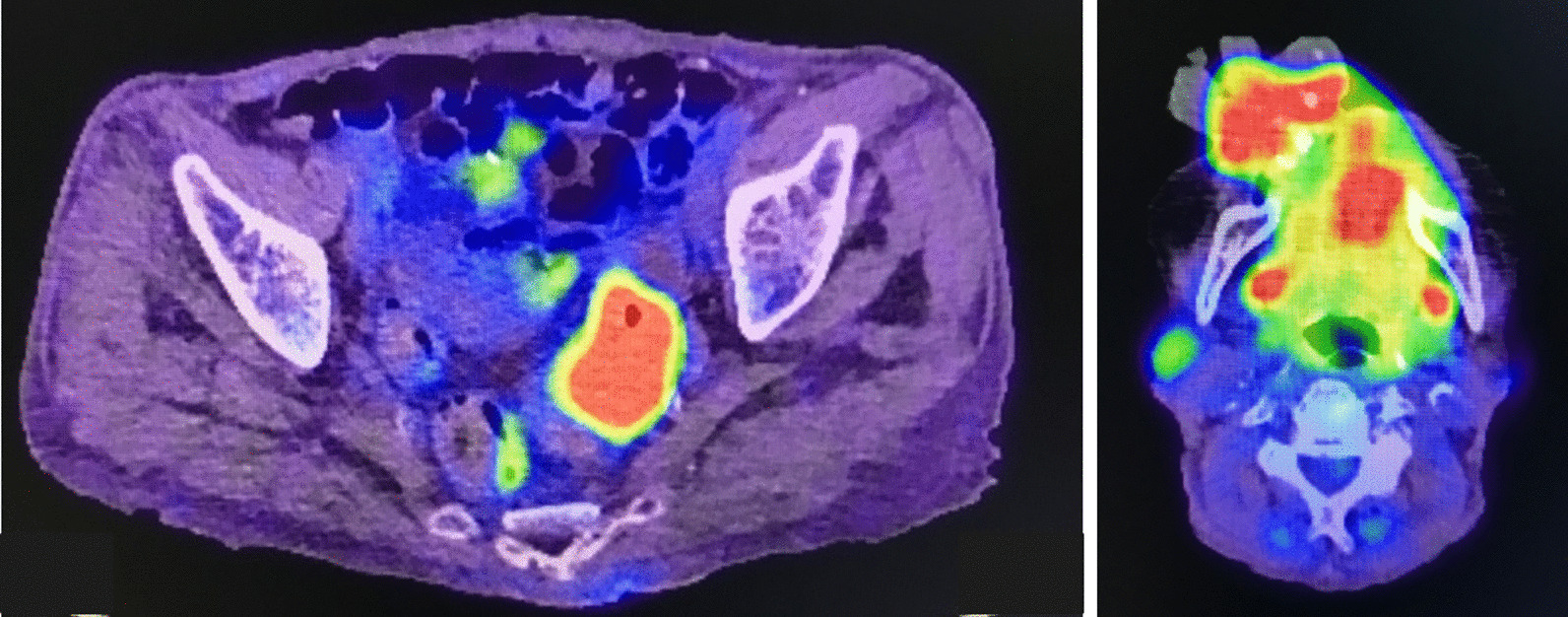
PET/CT reveals that the SUVmax of the sigmoid colon is 15.27, and that of the oral lesion is 14.99. There are no other obvious metastases

**Fig. 7.**
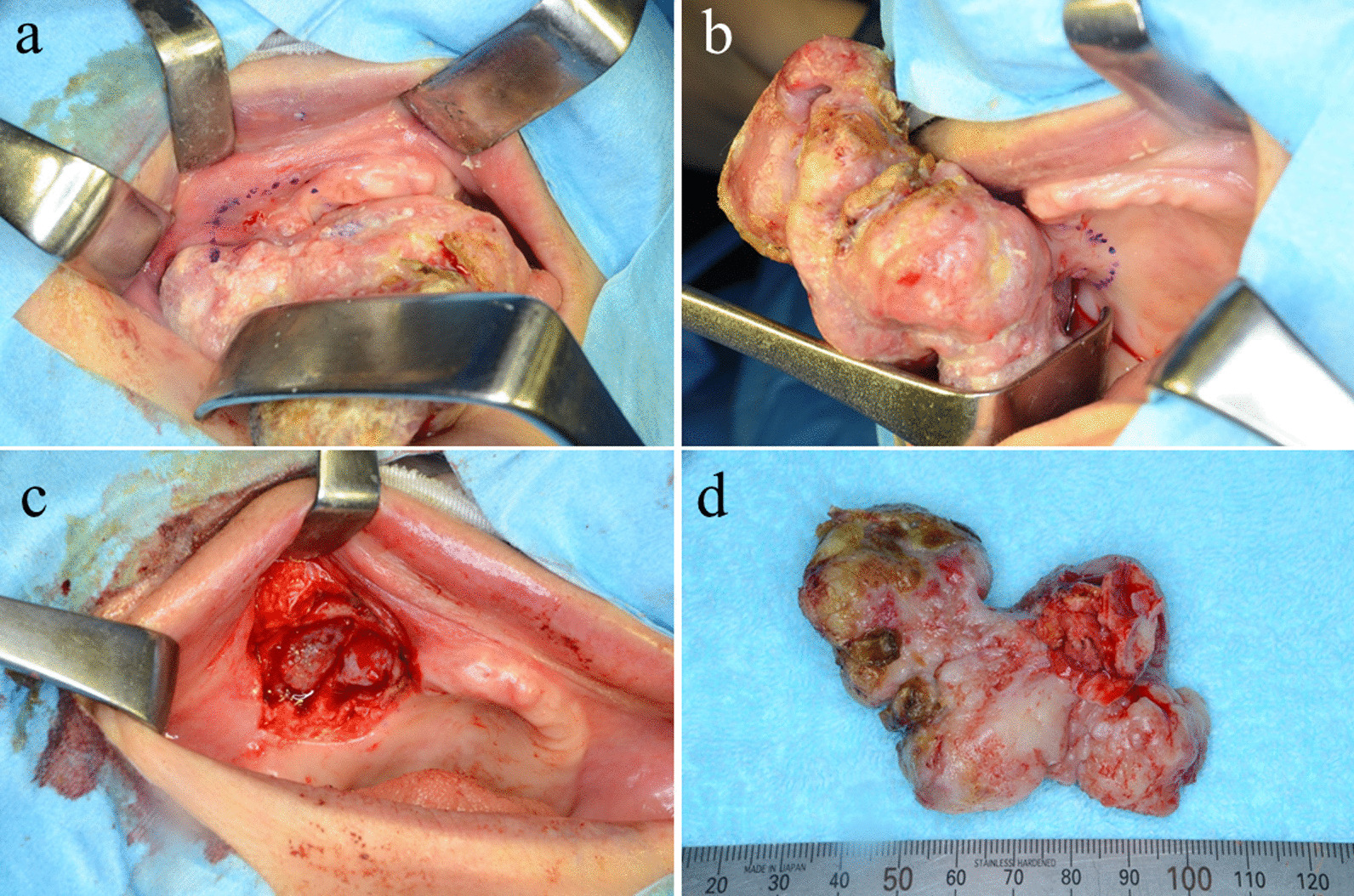
Operative findings. **a**, **b** The excision range was set with a safety margin of approximately 1 cm around the tumor. **c** Postoperative site of lesion. **d** Resected specimen

Histologically, proliferation of dysplastic squamous epithelia was observed (Fig. 5). We noted subepithelial mild dysplastic spindle-shaped cells and collagenous fibers, and scattered calcification and ossification were also observed (Fig. 5). Immunohistochemically, the spindle cells were negative for pan-cytokeratin (AE1/AE3), and nuclear translocation of β-catenin was not observed in the spindle cells (data not shown). Therefore, we excluded a diagnosis of carcinosarcoma and fibromatosis. However, we were unable to diagnose absolutely whether the dysplastic squamous epithelia were pseudocarcinomatous hyperplasia of the gingiva or well-differentiated squamous cell carcinoma. In addition, positron emission tomography with computed tomography (PET/CT) revealed that the maximum standard unit value (SUVmax) of the sigmoid colon and the oral lesion were 15.27 and 14.99, respectively, and there were no other obvious metastases (Fig. 6). Therefore, we performed tumorectomy under general anesthesia. The pedicle of the tumor was located at the right maxillary premolar area, and the tumor—including the tissue surrounding the lesion—was resected as one mass together with the periosteum (Fig. 7a–d). At that time, partial destruction of the maxillary bone was seen. The exposed bone surface was slightly curetted. After resection, the wounded area was covered with artificial dermis (TERDERMIS). Finally, tie-over dressing by gauze with ointment was performed.

**Fig. 8.**
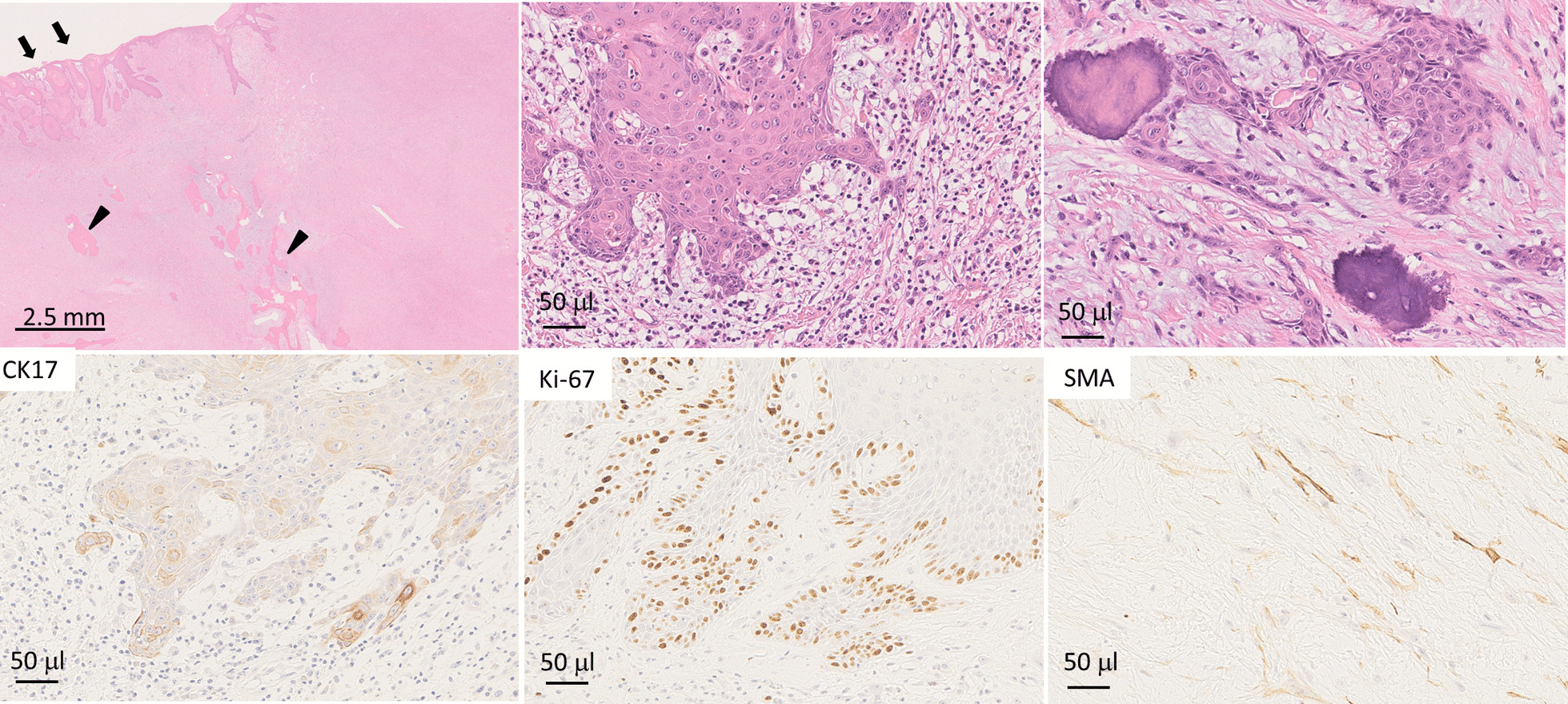
Histopathological findings (hematoxylin–eosin and immunohistochemical stain). The body of the tumor is composed of spindle-shaped cells proliferating with collagenous fiber, and marked ossification can be seen as well. Squamous cell carcinoma is found in the superficial part of the lesion (squamous cell carcinoma; arrow, ossification; arrowhead). Positive staining of CK17 and Ki67 observed in atypical squamous epithelia supports the diagnosis of squamous cell carcinoma, and focal SMA staining indicates myofibroblastic differentiation

The microscopic findings of the surgically removed tumor were similar to those of the biopsy specimen. The body of the tumor was composed of spindle-shaped cells that were proliferating with collagenous fiber, and scattered bone formation was also observed (Fig. 8). The destruction of the basement membrane by atypical squamous epithelia was observed in the surgically removed specimen, suggesting stromal invasion. In addition, immunohistochemical analysis revealed that the dysplastic squamous epithelia were positive for Ki67 and CK17, suggesting that they were squamous cell carcinoma, not pseudocarcinomatous hyperplasia. Furthermore, some spindle cells were positive for smooth muscle action (SMA), indicating myofibroblastic differentiation. Therefore, the epithelial component of the tumor was considered to be well-differentiated squamous cell carcinoma. Because most of the tumor was occupied by spindle-shaped cells and marked ossification histopathologically, we diagnosed the tumor as POF with squamous cell carcinoma (pT1N0M0).

**Fig. 9.**
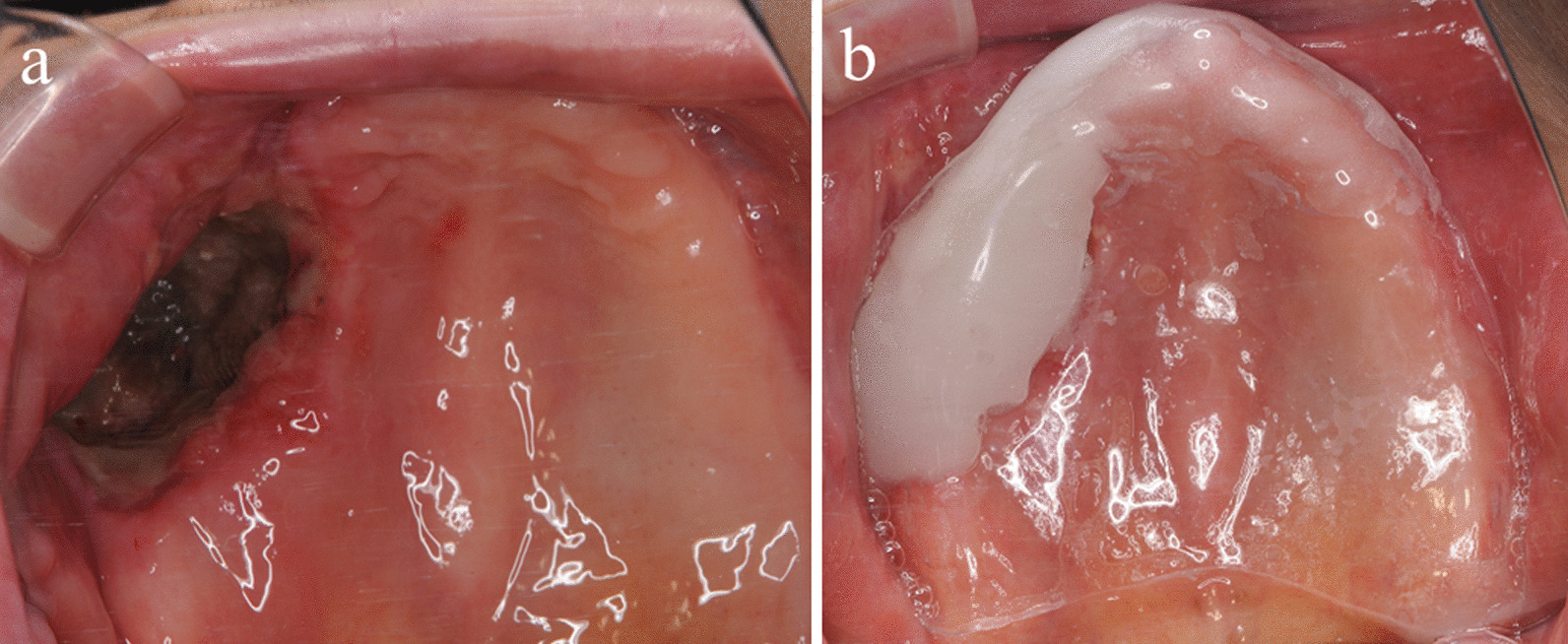
Clinical follow-up. **a** Seven days’ follow-up showing partial wound healing. **b** Covering with an oral appliance for protection

One week after surgery, we removed the gauze and covered the wound with an oral appliance for protection (Fig. 9a, b). There have been no signs of local recurrence or metastasis during follow-up as of 2 years after surgery.

## Discussion and conclusions

Exophytic gingival lesions consisting of cellular myofibroblast proliferation, including calcification [11, 12], have been described in the literature since the late 1940s. Various designations have been given to similar lesions, such as epulis, peripheral fibroma with calcification, and POF. They account for 3% of all oral tumors and 9.6% of all gingival lesions [12–14]. Among these, POF is a benign mesenchymal lesion, and Gardner [15] reinforced the notion that POF is not a peripheral variant of central ossifying fibroma, which is a true neoplasm but a distinct non-neoplastic inflammatory proliferation. Although the etiology and pathogenesis of POF remain unknown, it seems commonly to be a reactive condition and originate from cells in the periodontal ligament, probably related to trauma or local irritants [12, 14, 16, 17]. In our case, the patient probably still had a tooth at the time the mass developed. However, it is thought that she subsequently lost all of her teeth as she aged, thus resulting in the edentulous condition. Sacks *et al*.[10] also reported a case of giant POF in an edentulous patient. He noted that an edentulous state provided no resistance to the lesion’s proliferation. On the other hand, some authors have suggested that POFs are related to hormonal changes [1, 2, 18]. POF is usually small with an anterior maxillary presentation [5, 6]. It includes hard tissue in fibrous tissue, and the name “neoplastic lesion” has tended to be used frequently in Europe and America. Clinically, POFs are painless, solitary, exophytic, sessile, or pedunculated and more frequently found in females than in males [1–3], especially female patients in the second or third decade of life [3, 4]. In our case, the tumor was found in an elderly woman and was neglected for 14 years owing to the painless nature of the mass, which showed exophytic enlargement in the base of maxillary premolar region. Regarding radiographic evaluations, the presence of radiopacity, which is observed in soft tissues, represents the synthesis of bone (mature or immature), cementum, or calcifications, in various proportions. Immature woven bone is the most common type of such mineralization, but mature lamellar bone is seen as a form of maturation of the lesion. The present case included a large amount of mature bone, as the lesion had grown over a period of 14 years.

Histopathologically, POFs have a mineralized mass surrounded by a stroma of fibrocellular connective tissue. The definitive diagnosis depends on the results of the microscopic evaluation, especially with regard to the presence of bone or calcified materials as the key feature [11]. The hard tissues formed in POF are classified into trabecular bone, compact bone, lamellar bone, cementicles or ossicles, and dystrophic calcification [19–21]. In most cases, there is a mixture of these components. However, there are various theories regarding the origin of hard tissue formation, and no unified view has yet been obtained.

The immunohistochemical profile of POF has been poorly documented [4]. Some authors have already made use of anti-muscle actin antibody in their studies. For example, Marcos *et al*. [4] described four cases of POF, and their findings support a fibroblastic–myofibroblastic origin of the lesion, thus aiding in its differential diagnosis. In addition, Lázare *et al*.[22] used a panel of antibodies in 26 specimens that stained for smooth muscle actin in the spindle cell component in most cases and confirmed the myofibroblastic nature of the lesion. We used a panel of antibodies in the present specimen, which stained for smooth muscle actin in the spindle cell component. An imbalance in the structural and signaling properties of β-catenin often results in disease and deregulated growth connected to cancer and metastasis. In our case, the spindle cells were actin-positive and keratin-negative, suggesting that some of them had differentiated into myofibroblasts. However, since there was no nuclear migration of catenin, it was interpreted as reactive rather than fibromatosis.

The size of POF is often 10–20 mm [7, 9]. However, some lesions grow to more than 20 mm in size. Generally, POF grows slowly, and in today’s environment of strong investment in oral hygiene, it is extremely rare for them to grow to such a large size without resection. Some authors have occasionally described relatively large lesions in case reports of POF [8, 10, 23]. Although the most common site of POF is the anterior maxillary gingival area, there are few reports of it growing very large in the same region [10, 23, 24]. This is considered to be because the anterior area is easy to observe, and importance is attached to aesthetics, so treatment tends to be performed before the mass becomes large. Regarding the physiological function of the oral cavity, the tongue pressure is greater than the lip and buccal pressure, so the lesion seemed to have become relatively large and protruded from the oral cavity in our case. Giant POFs have several common clinical features, including large, atypical dimensions that may cause facial asymmetry and teeth displacement with no root resorption. Ectopic eruption, migration, and separation of teeth have been reported, as well as bone destruction [8, 24–26]. In our case, bone destruction was seen, although the patient was edentulous. Furthermore, giant lesions can impair speech, chewing, swallowing, aesthetics, and mouth closing. Similar to our case, most giant POFs are diagnosed in older adults [10, 24, 25]. Interestingly, while conventional POFs are more prevalent in females than in males, the giant type shows no sex predilection. Regarding the clinical appearance, giant POF commonly presents as an exophytic mass that can develop an ulcerated surface due to contact with the teeth on the opposite side of the mouth. The most common site of giant POF is the posterior mandible, although our present case does not fit this pattern description.

The treatment of POF requires total resection, including the periosteum and periodontal ligament, as well as all local etiological factors. Since recurrence can result from incomplete resection or failure to section the periodontal ligament, accurate resection is also required. The recurrence rate of POF is considered to be relatively high, ranging from 8% to 20% [9, 27]. Thus, strict postoperative follow-up is necessary to detect early recurrence. Although most literature supports the notion that the periodontal ligament is the origin of the lesion [12, 14, 16, 17, 22], there are reports of POF in edentulous patients aside from the present patient [1, 10].

Whether POF is a true neoplasm or a reactive proliferation is unclear. Therefore, POF is not included in the World Health Organization (WHO) 2017 Classification of Head and Neck Tumors. POF contains variable proportions of inflammatory cells, such as lymphocytes. Interestingly, although some giant POFs are characterized as neoplastic growths, to our knowledge, there have been no cases of POF with coincidental squamous cell carcinoma except for the present case. Because POF is a mesenchymal tumor, it was speculated that the squamous cell carcinoma in this case may have been caused by trauma as POF or may have been the result of exposure of the mucosa to ultraviolet rays from the sun or other stimulant. As for metastasis, the sigmoid colon carcinoma was an adenocarcinoma, and the oral lesion was a squamous cell carcinoma, suggesting that there was no mutual metastasis. In our case, immunohistochemical staining revealed partial p53 positivity (data not shown) as well as CK17 and Ki67 positivity. Moreover, hematoxylin–eosin staining also showed squamous cell carcinoma morphologically. However, the etiology of giant POF with squamous cell carcinoma is still not definite, and needs to be examined by accumulation of more cases in the future.

## Data Availability

The datasets created during and/or analyzed during this case are available from the corresponding author on reasonable request.

## Abbreviations

POF: Peripheral ossifying fibroma
CT: Computed tomography
PET/CT: Positron emission tomography with computed tomography

## References

[1] Kumar SK, Ram S, Jorgensen MG, Shuler CF, Sedghizadeh PP. Multicentric peripheral ossifying fibroma. J Oral Sci. 2006;48:239–43. 10.2334/josnusd.48.239 17220623

[2] Walters JD, Will JK, Hatfield RD, Cacchillo DA, Raabe DA. Excision and repair of the peripheral ossifying fibroma: a report of 3 cases. J Periodontol. 2001;72:939–44. 10.1902/jop.2001.72.7.939 11495143

[3] Yadav R, Gulati A. Peripheral ossifying fibroma: a case report. J Oral Sci. 2009;51:151–4. 10.2334/josnusd.51.151 19325214

[4] García de Marcos JA. Peripheral ossifying fibroma: a clinical and immunohistochemical study of four cases. J Oral Sci. 2010;52:95–9. 10.2334/josnusd.52.95 20339239

[5] Kenney JN, Kaugars GE, Abbey LM. Comparison between the peripheral ossifying fibroma and peripheral odontogenic fibroma. J Oral Maxillofac Surg. 1989;47:378–82. 10.1016/0278-2391(89)90339-x 2926546

[6] Zhang W, Chen Y, An Z, Geng N, Bao D. Reactive gingival lesions: a retrospective study of 2,439 cases. Quintessence Int. 2007;38:103–10. 17263149

[7] Stafne EC. Peripheral fibroma (epulis) that contains a cementum-like substance. Oral Surg Oral Med Oral Pathol. 1951;4:463–5. 10.1016/0030-4220(51)90172-7 14827538

[8] Poon CK, Kwan PC, Chao SY. Giant peripheral ossifying fibroma of the maxilla: report of a case. J Oral Maxillofac Surg. 1995;53:695–8. 10.1016/0278-2391(95)90174-4 7776054

[9] Farquhar T, Maclellan J, Dyment H, Anderson RD. Peripheral ossifying fibroma: a case report. J Can Dent Assoc. 2008;74:809–12. 19000465

[10] Sacks HG, Amrani S, Anderson K. “Gigantiform” peripheral ossifying fibroma: report of a case. J Oral Maxillofac Surg. 2012;70:2610–3. 10.1016/j.joms.2011.12.011 22310455

[11] Poonacha KS, Shigli AL, Shirol D. Peripheral ossifying fibroma: a clinical report. Contemp Clin Dent. 2010;1:54–6. 10.4103/0976-237X.62520 PMC3220071 22215935

[12] Verma E, Chakki AB, Nagaral SC, Ganji KK. Peripheral cemento-ossifying fibroma: case series literature review. Case Rep Dent. 2013;2013(1):930870. 10.1155/2013/930870 PMC3556846 23365762

[13] Chatterjee A, Ajmera N, Singh A. Peripheral cemento-ossifying fibroma of maxilla. J Indian Soc Periodontol. 2010;14:186–9. 10.4103/0972-124X.75915 PMC3100863 21760674

[14] Dahiya P, Kamal R, Saini G, Agarwal S. Peripheral ossifying fibroma. J Nat Sci Biol Med. 2012;3:94–6. 10.4103/0976-9668.95983 PMC3361788 22690061

[15] Gardner DG. The peripheral odontogenic fibroma: an attempt at clarification. Oral Surg Oral Med Oral Pathol. 1982;54:40–8. 10.1016/0030-4220(82)90415-7 6750498

[16] Cuisia ZE, Brannon RB. Peripheral ossifying fibroma—a clinical evaluation of 134 pediatric cases. Pediatr Dent. 2001;23:245–8. 11447957

[17] Luvizuto ER, Da Silva JB, Luvizuto GC, Pereira FP, Faco EF, Sedlacek P, Poi WR. Peripheral ossifying fibroma. J Craniofac Surg. 2012;23:e7-10. 10.1097/SCS.0b013e3182420729 22337469

[18] Moon WJ, Choi SY, Chung EC, Kwon KH, Chae SW. Peripheral ossifying fibroma in the oral cavity: CT and MR findings. Dentomaxillofac Radiol. 2007;36:180–2. 10.1259/dmfr/59377498 17463105

[19] Bhaskar SN, Jacoway JR. Peripheral fibroma and peripheral fibroma with calcification: report of 376 cases. J Am Dent Assoc. 1966;73:1312–20. 10.14219/jada.archive.1966.0375 5223950

[20] Buchner A, Ficarra G, Hansen LS. Peripheral odontogenic fibroma. Oral Surg Oral Med Oral Pathol. 1987;64:432–8. 10.1016/0030-4220(87)90148-4 3477764

[21] Zain RB, Fei YJ. Fibrous lesions of the gingiva: a histopathologic analysis of 204 cases. Oral Surg Oral Med Oral Pathol. 1990;70:466–70. 10.1016/0030-4220(90)90212-b 2120653

[22] Lazare H, Peteiro A, Perez Sayans M, Gandara-Vila P, Caneiro J, Garcia-Garcia A, Anton I, Gandara-Rey JM, Suarez-Penaranda JM. Clinicopathological features of peripheral ossifying fibroma in a series of 41 patients. Br J Oral Maxillofac Surg. 2019;57:1081–5. 10.1016/j.bjoms.2019.09.020 31601435

[23] Reddy V, Kv A, Wadhwan V, Venkatesh A. Giant peripheral ossifying fibroma of the posterior mandible—a rare case report. Iran J Pathol. 2017;12:397–401. PMC5844686 29563937

[24] Childers EL, Morton I, Fryer CE, Shokrani B. Giant peripheral ossifying fibroma: a case report and clinicopathologic review of 10 cases from the literature. Head Neck Pathol. 2013;7:356–60. 10.1007/s12105-013-0452-1 PMC3824796 23857548

[25] Célio-Mariano R, Oliveira M, de Carvalho Silva A. Large peripheral ossifying fibroma: clinical, histological, and immunohistochemistry aspects. A case report. Rev Esp Cir Oral Maxilofac. 2017;39:39–43.

[26] Freire AEN, da Silva VSA, Pereira AAC, Ribeiro Junior NV, de Carli ML, Sperandio FF, Hanemann JAC. Giant peripheral ossifying fibroma treated with piezosurgery and platelet-rich fibrin: a rare case report. Clin Adv Periodontics. 2019;9:15–9. 10.1002/cap.10042 31490037

[27] Trasad VA, Devarsa GM, Subba Reddy VV, Shashikiran ND. Peripheral ossifying fibroma in the maxillary arch. J Indian Soc Pedod Prev Dent. 2011;29:255–9. 10.4103/0970-4388.85837 21985885

